# BZW1 Drives Immune Evasion in Lung Adenocarcinoma via Ferroptosis Suppression

**DOI:** 10.1002/advs.202521885

**Published:** 2026-03-15

**Authors:** Linyao Zhao, Yue Peng, Qing Liang, Shi Liu, Yang Li, Lei Ma, Menghan Hu, Sujuan Zheng, Zhihua Liu, Shugeng Gao

**Affiliations:** ^1^ Department of Thoracic Surgery National Cancer Center/National Clinical Research Center for Cancer/Cancer Hospital Chinese Academy of Medical Sciences and Peking Union Medical College Beijing China; ^2^ Department of Thoracic Surgery Beijing Institute of Respiratory Medicine and Beijing Chao‐Yang Hospital Capital Medical University Beijing China; ^3^ State Key Laboratory of Molecular Oncology National Cancer Center/National Clinical Research Center for Cancer/Cancer Hospital Chinese Academy of Medical Sciences and Peking Union Medical College Beijing China

**Keywords:** BZW1, ferritinophagy, ferroptosis, immunotherapy, intracellular ferritin complex, lung cancer

## Abstract

Despite multiple therapeutic strategies have provided clinical benefit for certain subsets of non‐small cell lung cancer (NSCLC) patients, achieving durable treatment responses remains a significant challenge. Immunotherapy has shown clinical benefits in lung cancer patients, while the efficacy is not quite satisfactory, especially in patients with lung adenocarcinoma (LUAD). Ferroptosis, a form of programmed cell death driven by iron‐dependent lipid peroxidation, has recently emerged as a critical regulator of metabolic circuitry and anti‐tumor immunity. Here, we identify *BZW1* (Basic Leucine Zipper and W2 Domains 1) as a central regulator that promotes immune evasion through ferroptosis suppression in LUAD. Mechanistically, BZW1 attenuates ferroptosis via suppression of FTH1 degradation via autophagic degradation of NCOA4, the selective cargo receptor. Moreover, BZW1 competitively binds with NCOA4 and disrupts the binding of FTH1 and NCOA4, thus inhibiting ferritinophagy‐mediated ferritin degradation.  BZW1 attenuates ferroptosis and creates an immunosuppressive microenvironment by reducing immunogenic cell death and impairing T cell activation. Our findings establish BZW1 as a ferroptosis suppressor whose inhibition may synergize with immunotherapy in LUAD, highlighting the therapeutic potential of targeting the BZW1‐ferroptosis axis for lung cancer treatment.

## Graphical abstract

BZW1 attenuates ferroptosis by competitively binding NCOA4 and suppress ferrtinophagy‐mediated iron release. The depletion of BZW1 triggers lipid peroxidation through iron homeostasis. Extracellularly, BZW1 attenuates immunogenic cell death and reinvigorates cytotoxic T‐cell responses. Combining BZW1 targeting with anti‐PD‐1 therapy synergistically enhances antitumor immunity, and these regulatory systems position BZW1 as a promising therapeutic target for sensitizing lung cancer to immunotherapy.

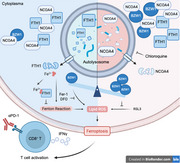

## 1 Introduction

Lung cancer is responsible for the cancer burden worldwide, and maintains as the leading cause (18.7%) of cancer‐related deaths worldwide, with an estimated 2.5 million new cases in 2022 [1]. Non‐small‐cell histology dominates, with adenocarcinoma (∼50%) and squamous cell carcinoma (∼20–30%) constituting the bulk of cases. Despite the introduction of innovative therapeutic options and neoadjuvant therapy, including immune checkpoint blockers (ICBs) and targeted therapies, which have transitioned to earlier stages, the 5‐year average survival rate of patients with lung cancer remains suboptimal [2]. Landmark clinical trials, including CheckMate 816 and KEYNOTE‐671, have revealed that only approximately 20% of patients with resectable non‐small cell lung cancer achieve pathological complete response (pCR) in neoadjuvant immunotherapy, and these patients have shown sustained better survival, which emphasizes the advantage of a good immune response [3, 4]. Therefore, it shed lights on improving the immune response to anti‐PD‐1, especially in patients with no major pathological response (NMPR). The marked heterogeneity in treatment outcomes underscores an urgent need to elucidate the biological determinants underlying differential responses to neoadjuvant immunotherapy [5]. The tumor immune microenvironment (TIME) has emerged as a critical mediator of therapeutic efficacy, with its cellular composition, spatial organization, and functional state, which could be predictors of pathological response in neoadjuvant therapies. However, the molecular regulators of these immunomodulatory programs remain inadequately characterized. Delineating key oncogenic drivers and their mechanistic interplay with TIME remodeling is therefore imperative for developing targeted combination therapies, particularly in the context of rapidly evolving treatment paradigms for non‐small cell lung cancer [6, 7].

*BZW1* (basic leucine zipper and W2 domains 1), a basic leucine zipper protein, is emerging as a pivotal pan‐cancer driver and has been identified as an important driver of tumor progression. Multi‐omics analyses reveal its consistent over‐expression in lung adenocarcinoma, gastric, pancreatic, and salivary mucoepidermoid carcinomas, where high levels independently predict poor overall and disease‐free survival [8, 9, 10, 11]. Mechanistically, BZW1 mainly exerts pleiotropic oncogenic functions by converging on proliferation and epithelial‐mesenchymal transition (EMT). Moreover, bioinformatic analysis reported that BZW1 possibly shapes the TIME and creates an immunosuppressive niche that limits the efficacy of ICBs in pancreatic cancer and glioblastoma, while the effect and mechanism of *BZW1* on TIME were not elucidated [8].

As the name indicates, ferroptosis is ignited by iron‐catalyzed lipid peroxidation, and ferritin is the primary iron storage complex [12, 13]. Iron metabolism is a core process in ferroptosis, during which the labile iron pool generates reactive oxygen species (ROS) through the Fenton reaction. Ferritin is the main intracellular storage form of iron, which can store iron ions and prevent ROS generation. Ferritin heavy chain 1 (FTH1) is the key component of ferritin, which could sequester excess iron in a non‐toxic, bioavailable form [14, 15]. Ferritinophagy, a selective form of autophagy, has emerged as a fundamental cellular process that regulates iron metabolism by mediating the degradation of ferritin [16]. The process is mediated by the cargo receptor nuclear receptor coactivator 4 (NCOA4), which specifically targets FTH1 to autophagosomes for lysosomal degradation [16, 17, 18]. Recent advances have shown that ferroptosis could be suppressed via the disruption of the complex in neurological diseases and sepsis, but whether the complex could be disrupted in cancer, leading to ferroptosis suppression, was not fully elucidated [19, 20].

Emerging evidence has established a crucial interface between ferroptosis and antitumor immunity [21, 22, 23, 24, 25]. As the primary mediators of cellular immunity, CD8^+^ cytotoxic T lymphocytes eliminate malignant cells through the secretion of effector molecules, including IFN‐γ. However, tumor cells develop sophisticated adaptive mechanisms to resist ferroptotic death, constituting an important pathway of immune escape [25]. These resistance mechanisms include enhanced antioxidant capacity through NRF2 activation, upregulation of GPX4, and modulation of iron metabolism through ferritinophagy inhibition [15, 26, 27]. By evading this critical cell death pathway, tumor cells resist the effector function of tumor‐infiltrating lymphocytes and establish an immunosuppressive microenvironment for immune evasion [24]. Understanding the molecular contributors to ferroptosis resistance may therefore reveal novel therapeutic vulnerabilities.

Here we identified BZW1 as a significant regulator that suppresses ferroptosis and drives immune evasion. Mechanistically, BZW1 promotes the autophagic degradation of NCOA4 and physically disrupts the NCOA4‐FTH1 complex via a competitive binding with NCOA4, preventing NCOA4‐mediated autophagic degradation of ferritin and dampening ferritinophagy; thus, restraint on iron release reinforces ferroptosis resistance. Concurrently, BZW1 remodels the tumor immune microenvironment toward a suppressive state, further shielding malignant cells from immune attack. By co‐targeting these targets and immune checkpoints, our findings open new avenues for enhancing immunotherapy and overcoming immunotherapy resistance in NSCLC.

## 2 Results

### 2.1 BZW1 is Highly Expressed in Lung Adenocarcinoma and Correlates with Poor Prognosis

**FIGURE 1:**
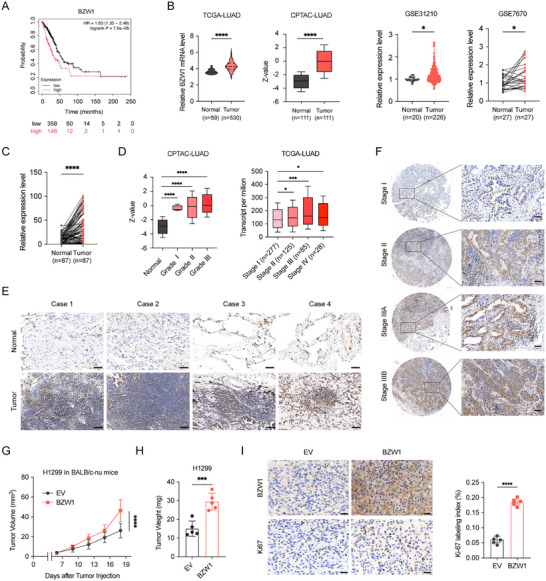
BZW1 is highly expressed in lung adenocarcinoma and correlates with poor prognosis. (A) Kaplan‐Meier survival analysis of lung adenocarcinoma patients stratified by BZW1 expression levels. (B) BZW1 mRNA expression and protein expression in lung adenocarcinoma tumor tissues and normal lung tissues from public databases (TCGA, CPTAC) and large scale datasets. (C) Tissue microarray (TMA) analysis of BZW1 protein expression in paired tumor and adjacent normal tissues. (D) BZW1 expression levels across different tumor grades and clinical stages. (E) Representative immunohistochemical staining of BZW1 in normal lung and lung cancer tissues. Scale bar: 50 µm. (F) IHC analysis of BZW1 expression in tumors of different stages. Scale bar: 50 µm. (G) Tumor growth curves in nude mouse xenograft models with BZW1 overexpression (n = 5). (H) Tumor weights from xenograft models at endpoint. I) Representative IHC staining of BZW1 and Ki‐67 in xenograft tumor sections and quantitative analysis. The data are presented as the mean ± s.e.m. ^*^ *p* < 0.05, ^**^ *p* < 0.01, ^***^ *p* < 0.001, ^****^ *p* < 0.0001.

To confirm the oncogenic role of BZW1 in NSCLC, we first performed survival analysis, which showed that high BZW1 expression was significantly associated with poor patient prognosis (Figure 1A) [28]. Analysis of large‐scale transcriptomic databases demonstrated that BZW1 expression is elevated in tumor tissues compared to normal tissues in lung cancer and pan‐cancer (Figure 1B; Figure S1A–C). Validation in a paired dataset and in‐house cohort tissue microarray (TMA) showed an elevated expression level of BZW1 in tumor tissue compared to adjacent normal tissue. (Figure 1C). Moreover, BZW1 expression showed a progressive increase with disease advancement, with significantly higher levels in advanced‐stage tumors compared to early‐stage lesions (Figure 1D). Immunohistochemistry staining results in paired samples and TMA are consistent as well (Figure 1E,F). To functionally characterize BZW1's oncogenic properties, we established xenograft models in nude mice. BZW1‐overexpressing tumors exhibited significantly accelerated growth kinetics compared to control groups (Figure 1G,H). Consistent with the observed tumor‐promoting phenotype, immunohistochemical analysis revealed enhanced Ki‐67 staining in BZW1‐overexpressing tumors, indicating increased proliferative activity (Figure 1I).

### 2.2 BZW1 Shapes an Immunosuppressive Niche in the Lung Tumor Microenvironment

**FIGURE 2:**
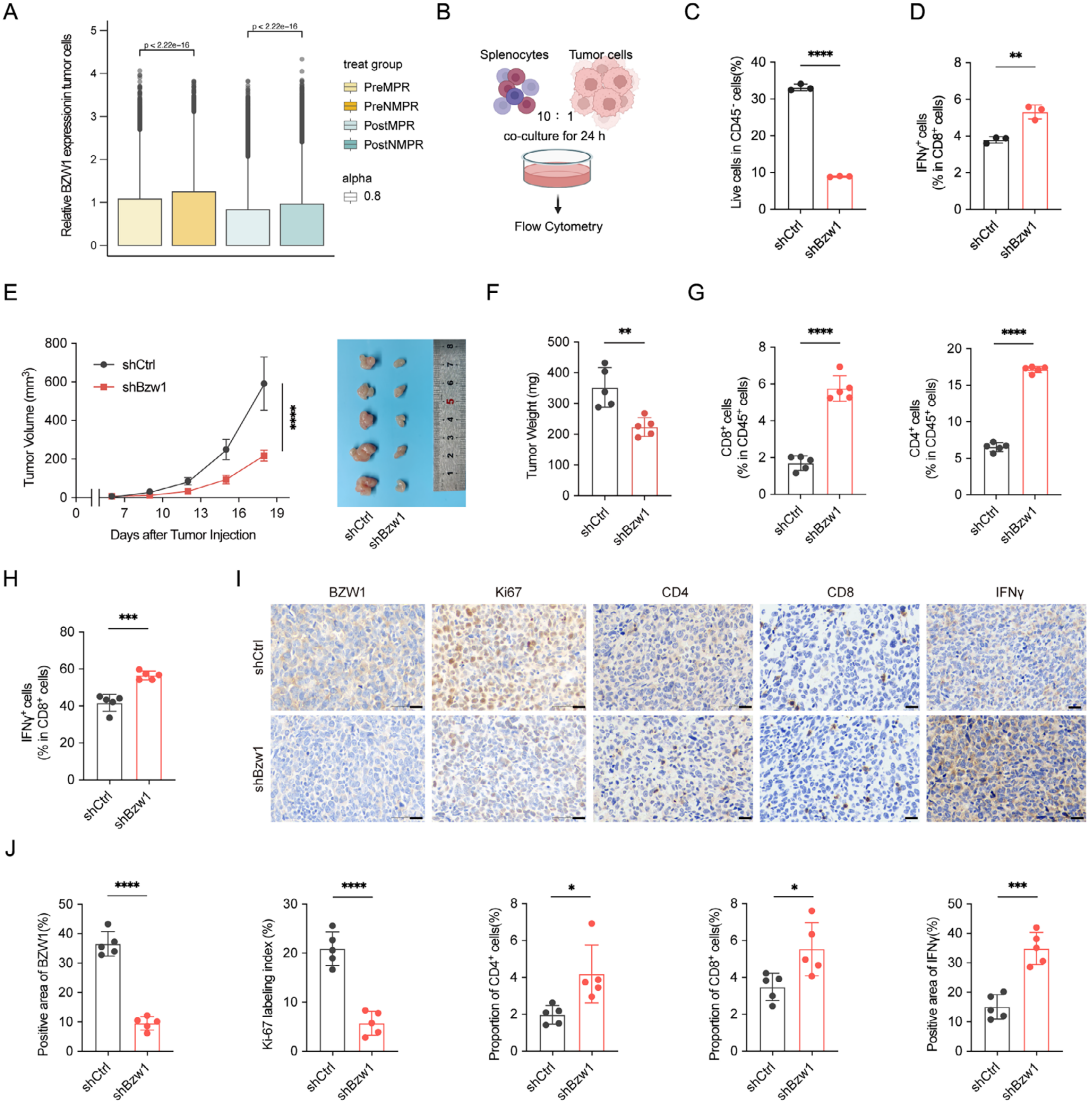
BZW1 shapes an immunosuppressive microenvironment in lung cancer. (A) BZW1 expression in tumor cells from MPR(n = 6) and NMPR(n = 13) patients following neoadjuvant immunotherapy. (B) Schematic diagram of the tumor cell‐splenocyte co‐culture system. (C) Quantification of splenocyte‐mediated tumor cell killing using Zombie Aqua viability staining (n = 3). (D) Frequencies of IFN‐γ^+^ CD8^+^ T cells in the co‐culture system (n = 3). (E) Tumor growth curves and representative images of resected tumors from each experimental group. (F) Tumor weights at the experimental endpoint (n = 5). (G) Flow cytometric analysis of CD4^+^ and CD8^+^ T cell infiltration in tumors (n = 5) (H). Frequencies of IFN‐γ^+^ CD8^+^ T cells in tumors (n = 5). (I, J) Representative immunohistochemical staining and quantitative analysis of BZW1, Ki‐67, CD4, CD8 and IFN‐γ in tumor sections. The data are presented as the mean ± s.e.m. ^*^ *p* < 0.05, ^**^ *p* < 0.01, ^***^ *p* < 0.001, ^****^ *p* < 0.0001.

To confirm if BZW1 could affect the response to immune checkpoint blockade, we first conducted bioinformatic analysis of the TCGA‐LUAD dataset (Figure S2A) [29], which showed that BZW1 shapes an immunosuppressive environment in lung cancer. To further confirm this effect in immunotherapy, we validate the expression of BZW1 in pre‐treatment and post‐treatment biopsies in an immunotherapy cohort. Results showed that BZW1 expression level in tumor cells is lower in patients with major pathological response (MPR), indicating that BZW1 expression level could possibly affect the therapeutic response to immunotherapy (Figure2A) [6]. To further investigate tumor‐immune cell crosstalk in lung cancer, we established a co‐culture system combining Lewis lung carcinoma (LLC) cells expressing shCtrl or shBzw1 with mouse splenocytes (Figure 2B). Flow cytometric analysis demonstrated that Bzw1 knockdown significantly enhanced splenocyte‐mediated tumor cell killing (Figure 2C; Figure S2B). Since cytotoxic T cells are the primary effector in TIME, we assessed the proportion of IFN‐γ^+^ CD8^+^ T cells, results showed that IFN‐γ^+^ CD8^+^ T cells were increased in splenocytes in shBzw1 group, indicating that Bzw1 modulates the tumor immune microenvironment by cytotoxic T cell activation (Figure 2D). To further validate these findings in vivo, we performed a xenograft assay, and results showed that Bzw1 knockdown markedly suppressed tumor growth (Figure 2E,F). Flow cytometric analysis showed that Bzw1 knockdown enhanced infiltration of both CD4^+^ and CD8^+^ T cells and increased the frequency of IFN‐γ^+^ CD8^+^ T cells within tumors (Figure 2G,H). Immunohistochemistry results were consistent as well (Figure 2I,J). We also observed an elevated frequency of Th1 cells and a reduction in myeloid‐derived suppressor cells (MDSCs) and M2‐like macrophages (Figure S2C). Together, these results demonstrate that BZW1 shapes an immunosuppressive TIME.

### 2.3 BZW1 Inhibited Death of Lung Cancer Cells in a Ferroptosis‐Dependent Manner

**FIGURE 3:**
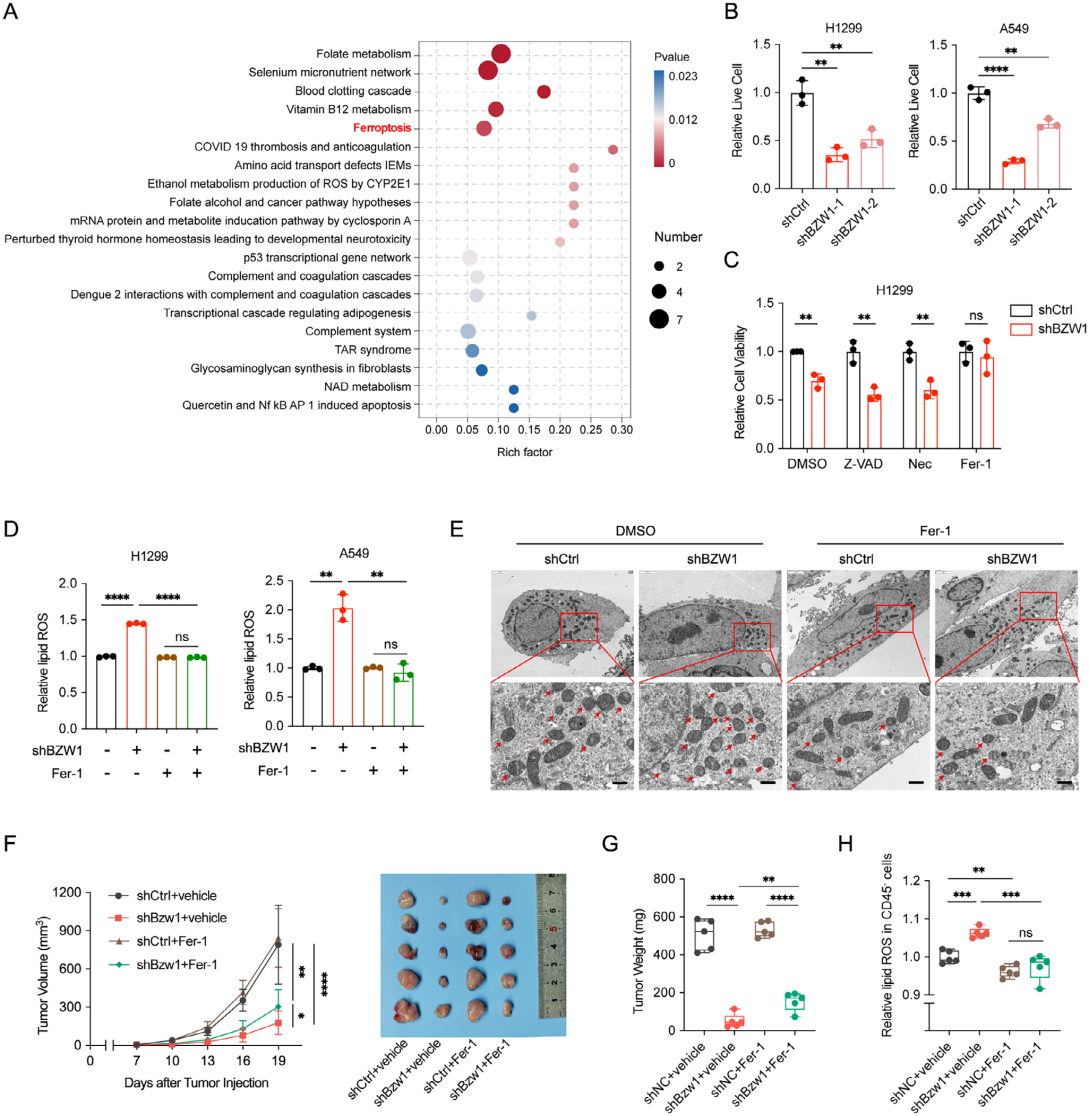
BZW1 suppresses ferroptosis in lung cancer cells. (A) Wikipathway enrichment of proteomics‐derived DEGs in BZW1‐knockdown H1299 cells. (B) Viability of H1299 and A549 cells with modulated BZW1 expression, measured by CCK‐8 assay (n = 3). (C) Cell viability of BZW1‐knockdown cells treated with Z‐VAD (20 µM), necrostatin‐1 (Nec‐1, 20 µM), or ferrostatin‐1 (Fer‐1, 20 µM), assessed by CCK‐8 assay (n = 3). (D) Lipid reactive oxygen species measured by C11‐BODIPY 581/591 staining in BZW1‐knockdown cells treated with Fer‐1 (5 µM, 24 h) (n = 3). (E) Representative TEM images of mitochondrial ultrastructure. Scale bar: 100 nm. (F) Tumor growth curves and representative tumor images from xenograft models (n = 5). (G) Tumor weights at endpoint (n = 5). (H) Lipid reactive oxygen species in xenografts assessed by C11‐BODIPY staining (n = 5). The data are presented as the mean ± s.e.m. ^*^ *p* < 0.05, ^**^ *p* < 0.01, ^***^ *p* < 0.001, ^****^ *p* < 0.0001.

To investigate the oncogenic role of BZW1 in lung cancer, we conducted transcriptomic and proteomic profiling in H1299 BZW1‐knockdown cells. Enrichment analysis of the multi‐omics data suggested that BZW1 may influence cellular processes through the regulation of ferroptosis (Figure 3A; Figure S3A). CCK‐8 assay revealed that knockdown of BZW1 decreased the cell viability of lung cancer cells (Figure 3B). Moreover, we treated BZW1‐knockdown cells with the ferroptosis inhibitors ferrostatin‐1 (Fer‐1), necroptosis inhibitor necrostatin‐1 (NEC), and apoptosis inhibitor Z‐VAD‐FMK. Cell viability assays showed that Fer‐1 effectively rescued cell death induced by BZW1 knockdown, indicating that BZW1 attenuates ferroptosis in lung cancer cells (Figure 3C; Figure S3B). To further verify this regulation, we assessed the lipid ROS in BZW1 knockdown cells with treatment of Fer‐1 for 24 h, and results showed that the elevated lipid ROS induced by BZW1 knockdown could be rescued by Fer‐1 (Figure 3D), and TEM results and MDA assessment showed similar results (Figure 3E; Figure S3C,D). Furthermore, we establish an in vivo rescue model to verify this ferroptosis‐dependent manner. The results showed that Fer‐1 treatment markedly attenuated the tumor‐suppressive effect of BZW1 knockdown (Figure 2F,G), and the elevated lipid peroxidation levels and MDA levels in tumor cells were reversed as well (Figure 2H; Figure S3E). Moreover, we assessed the proliferation and lipid ROS in BZW1 overexpressing cells treated with RSL3, results showed that BZW1 could resist RSL3‐induced ferroptosis (Figure S3F–I). Collectively, these data demonstrate that BZW1 regulates lung cancer progression through ferroptosis.

### 2.4 BZW1 Inhibits Ferroptosis Lung Cancer Cells via Intracellular Iron Homeostasis

**FIGURE 4:**
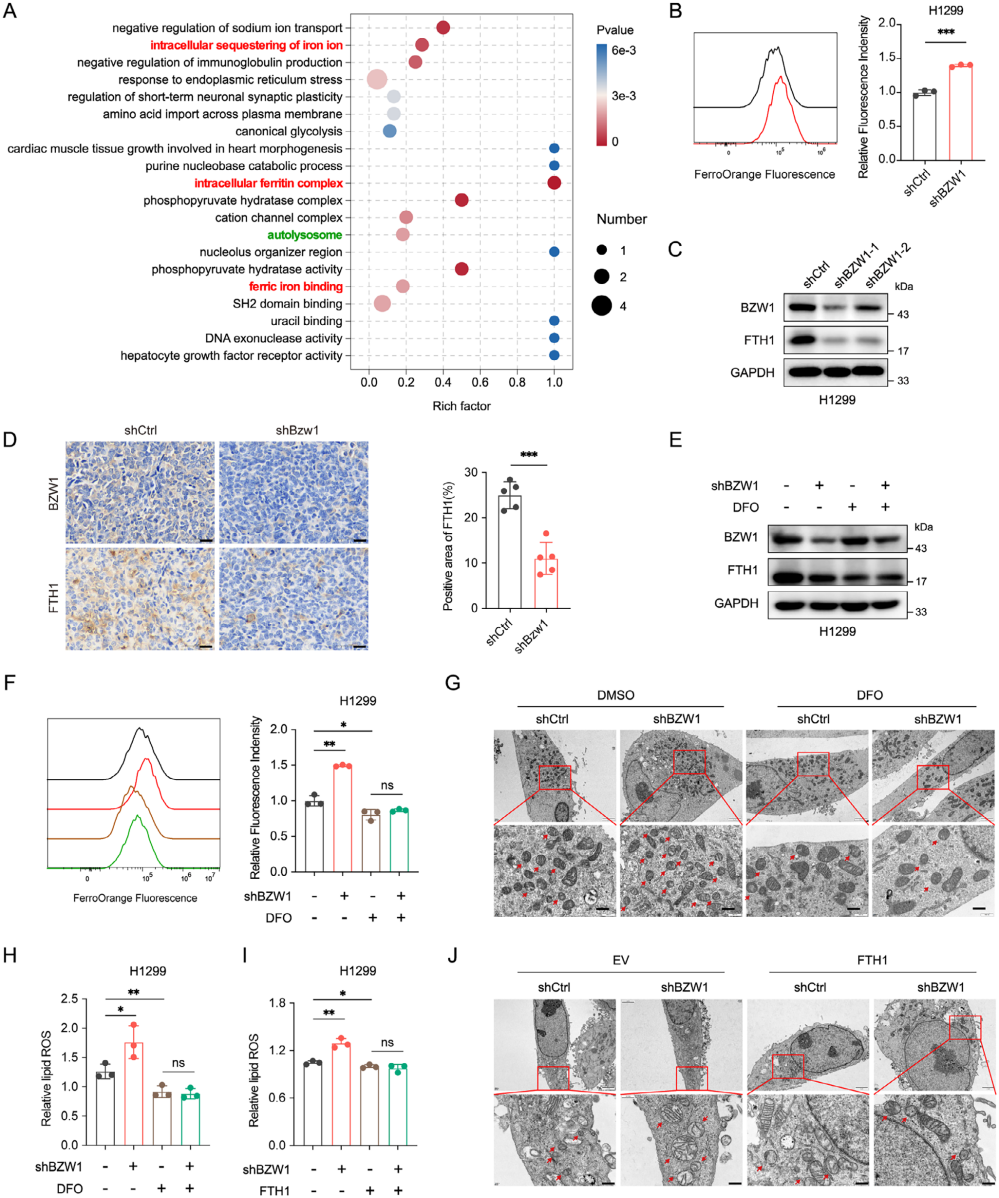
BZW1 regulates ferroptosis through modulation of the labile iron pool. (A) Gene Ontology (GO) enrichment analysis of differentially expressed proteins identified by proteomic sequencing in H1299 cells following BZW1 knockdown. (B) Intracellular Fe^2^^+^ levels measured by FerroOrange staining (n = 3). (C) Western blot analysis of FTH1 protein expression. (D) Representative immunohistochemical staining of FTH1 in xenograft tumors derived from the indicated cell lines. (E) Western blot analysis of FTH1 protein expression in indicating groups. (F) Lipid reactive oxygen species assessed by C11‐BODIPY 581/591 staining in indicated groups (n = 3). (G) Representative TEM images of mitochondrial ultrastructure. Scale bar: 100 nm. (H, I) Lipid reactive oxygen species assessed by C11‐BODIPY 581/591 staining in indicated groups (n = 3). (J) Representative TEM images of mitochondrial ultrastructure. Scale bar: 100 nm. Data are presented as mean ± SEM, ^*^ *p* < 0.05, ^**^ *p* < 0.01, ^***^ *p* < 0.001, ^****^ *p* < 0.0001.

To elucidate the regulatory mechanism of BZW1 on ferroptosis, we conducted Gene Ontology (GO) enrichment analysis. The results indicated that BZW1 is potentially related to intracellular iron ion sequestration and ferritin complex (Figure 4A). Therefore, we first assessed Fe^2+^ levels, and results showed that the intracellular Fe^2+^ level is increased in BZW1‐knockdown cells (Figure 4B; Figure S4B) and decreased in BZW1‐overexpressing cells (Figure S4C), indicating that BZW1 affects intracellular iron ion. Given that FTH1 is the core component of the ferritin complex, we examined FTH1 protein expression in both BZW1‐overexpressing and BZW1‐knockdown cells. Western blot and IHC staining results showed that FTH1 levels were positively correlated with BZW1 expression (Figure 4C–E; Figure S4D), suggesting that BZW1 regulates iron storage via FTH1. To further confirm that BZW1 regulates ferroptosis via iron homeostasis, we treated cells with the iron chelator deferoxamine (DFO). Results showed that DFO could chelate intracellular labile iron (Figure 4E,F; Figure S4E,F). Moreover, the elevated lipid ROS could be rescued by DFO (Figure 4H, Figure S4H), and TEM results are consistent as well (Figure 4G; Figure S4G). To further verify this iron‐dependent mechanism, we transfected BZW1 knockdown cells with FTH1 overexpressing plasmids, and the results showed that FTH1 overexpression could reverse the elevated lipid ROS (Figure 4I; Figure S4I). TEM results are similar as well (Figure 4J; Figure S4J). Together, these findings establish that BZW1 maintains iron homeostasis in lung cancer cells, and its loss leads to iron overload, thereby promoting ferroptotic cell death, indicating that this regulation is Fe^2+^‐dependent. All these results suggest that BZW1 regulates ferroptosis via the intracellular iron homeostasis.

### 2.5 BZW1 Regulates Intracellular Ferritin Complex via Ferritinophagy

**FIGURE 5:**
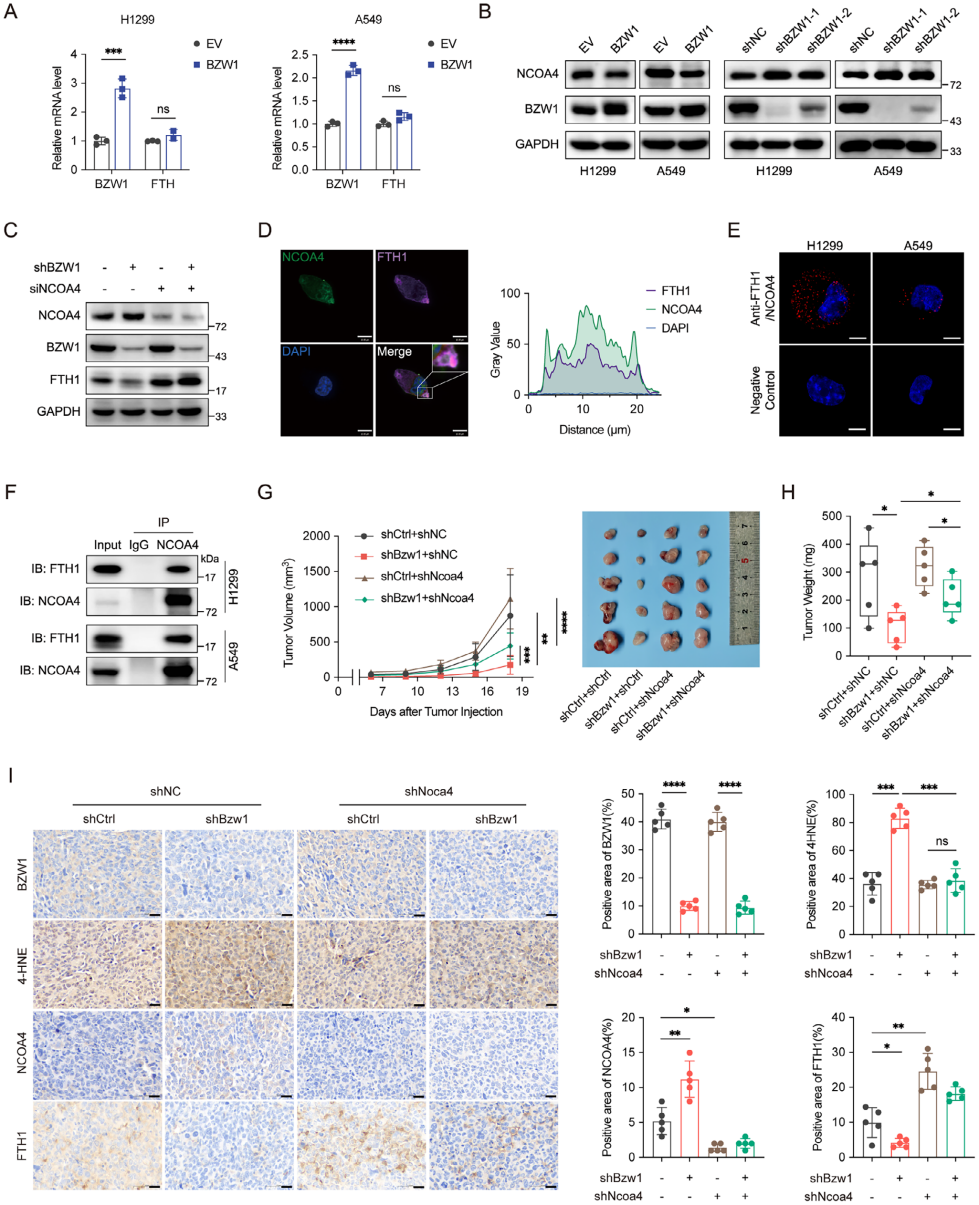
BZW1 modulates the labile iron pool through ferritinophagy regulation. (A) qRT‐PCR analysis of FTH1 mRNA expression in indicated experimental groups. (B) Western blot analysis of NCOA4 protein levels under indicated conditions. (C) Westernblot analysis of proteins in ferritinophagy pathways. (D) Immunofluorescence imaging of NCOA4 (green), FTH1 (purple), and DAPI‐stained nuclei (blue) in H1299 cells (50 µM). Scale bar: 10 µm. (E) PLA spots (red) assessment in H1299 and A549 cells. (F) Co‐IP assays demonstrating the interaction between FTH1 and NCOA4. (G) Tumor growth curves and representative tumor images from xenograft models. (H) Tumor weights at experimental endpoint (n = 5). (I) Representative immunohistochemical staining and analysis of BZW1, 4HNE, NCOA4, and FTH1 in xenograft tumors from indicated cell lines (n = 5). Data are presented as mean ± SEM, ^*^ *p* < 0.05, ^**^ *p* < 0.01, ^***^ *p* < 0.001, ^****^ *p* < 0.0001.

Given the results that BZW1 regulates ferroptosis through iron homeostasis, we focused on the regulation of ferritin, the primary iron depot. BZW1 overexpression elevated FTH1 protein abundance without altering its mRNA, indicating post‐transcriptional control (**Figure** **5** **A**). GO enrichment analysis revealed that autolysosome is possibly involved (Figure 4A), and ferritinophagy is the classical way of ferritin degradation; therefore, we evaluated the expression level of NCOA4, the selective cargo receptor of FTH1. Western blotting analysis revealed that NCOA4 is negatively correlated with BZW1 (Figure 5B). Western blot analysis revealed that the decreased FTH1 level in BZW1 knockdown cells could be reversed with NCOA4 knockdown, which confirms the regulatory role of NCOA4. Since NCOA4 is the canonical cargo receptor that escorts FTH1 to autolysosomes, we confirmed their co‐localization in H1299 cells by confocal immunofluorescence (Figure 5D). Direct association of FTH1 and NCOA4 was further verified through PLA assay and co‐immunoprecipitation (Figure 5E,F; Figure S5B). Xenograft assay results showed that NCOA4 knockdown could reverse the inhibitory effect of tumor growth of BZW1 knockdown (Figure 5G,H). Immunohistochemistry staining showed that knockdown of NCOA4 could reverse the declined expression level of FTH1 and BZW1 knockdown‐induced ferroptosis(Figure 5I). All these results showed that BZW1 regulates ferroptosis in a ferritinophagy‐dependent manner.

### 2.6 BZW1 Interrupts NCOA4‐FTH1 Complex via Direct Binding with and Autophagic Degradation of NCOA4

**FIGURE 6:**
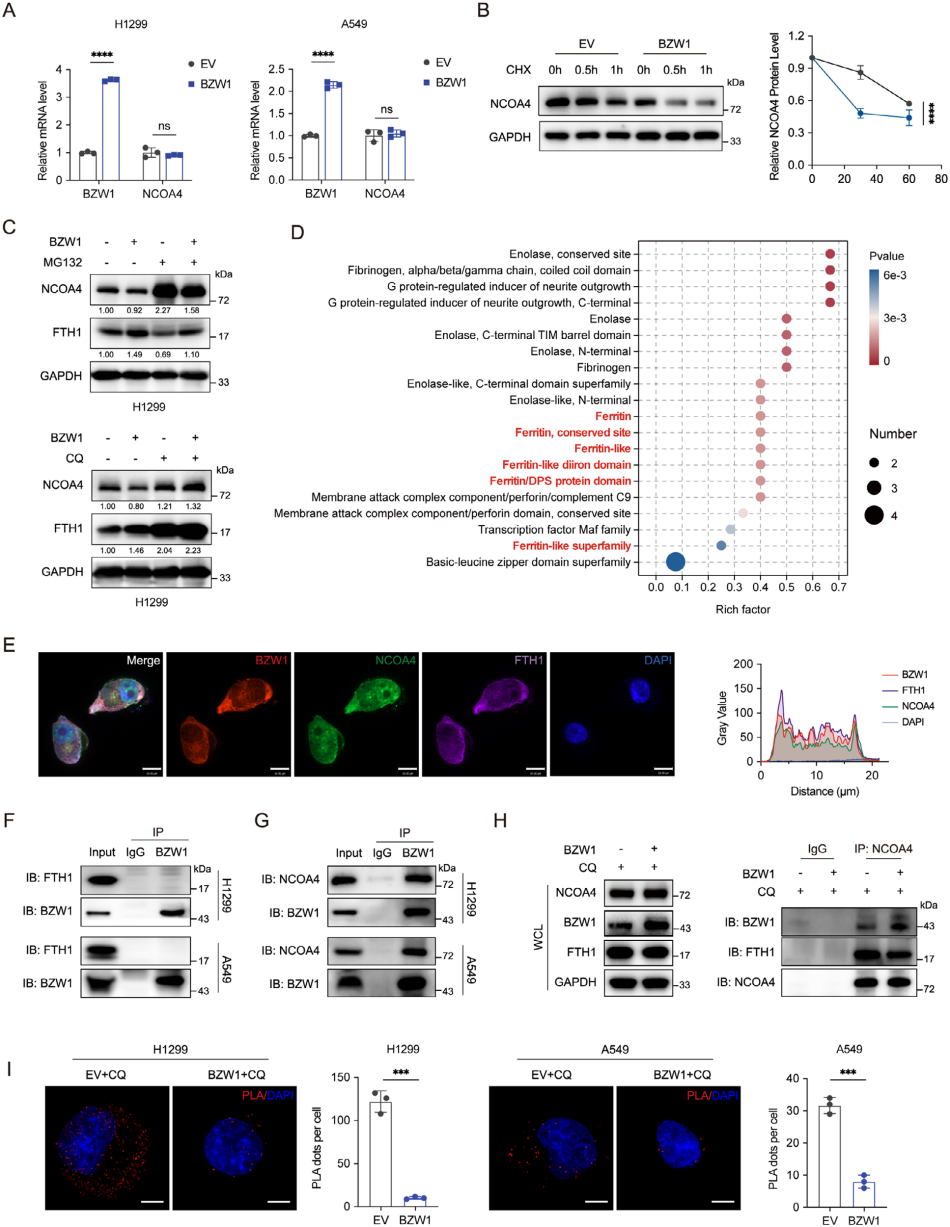
BZW1 disrupts the NCOA4‐FTH1 complex through direct binding to NCOA4. (A) qRT‐PCR analysis of NCOA4 mRNA expression in indicated cell lines. (B) Western blot analysis of NCOA4 protein degradation kinetics in cells treated with cycloheximide (100 µg/mL). (C) NCOA4 protein stability assessment in cells treated with MG132 (10 µM) and CQ (50 µM). (D) Protein domain enrichment analysis of differentially expressed proteins from proteomic sequencing in BZW1‐knockdown H1299 cells. (E) Confocal microscopy and colocalization analysis of BZW1 (red), NCOA4 (green), FTH1 (purple), and DAPI‐stained nuclei (blue). Scale bar: 10 µm. (F, G) Co‐IP assays analyzing BZW1‐FTH1‐NCOA4 protein interactions. (H) Co‐IP assay analyzing the effect of BZW1 on FTH1‐NCOA4 protein interactions. (I) PLA spots (red) assessment in H1299 and A549 cells. Data are presented as mean ± SEM, ^*^ *p* < 0.05, ^**^ *p* < 0.01, ^***^ *p* < 0.001, ^****^ *p* < 0.0001.

To elucidate the molecular mechanism by which BZW1 regulates NCOA4, we initially assessed NCOA4 transcript levels and observed that BZW1 downregulates NCOA4 protein expression without altering its mRNA abundance, suggesting post‐translational regulation (Figure 6A). We subsequently employed cycloheximide (CHX), MG132, and chloroquine (CQ) to interrogate the degradation pathway. The data demonstrate that BZW1 promotes autophagic degradation of NCOA4, as evidenced by the recovery of NCOA4 protein levels upon autophagy inhibition (Figure 6B,C; Figure S6A,B). Western blot analysis showed that autophagy was activated when BZW1 was overexpressed (Figure S6C), which confirms the autophagic degradation effect. Confocal microscopy results showed that the expression change of NCOA4 and FTH1 induced by BZW1 was reversed by CQ treatment (Figure S6D). To explore the possible protein‐protein interaction, we conducted domain enrichment analysis, which showed that multiple functional domains associated with ferritin complex and iron metabolism were enriched (Figure 6D). Immunofluorescence staining showed co‐localization among BZW1, NCOA4, and FTH1 (Figure 6E), while co‐IP assay results showed no direct binding between BZW1 and FTH1 (Figure 6F; Figure S6E), prompting us to investigate if BZW1 interferes with the NCOA4–FTH1 complex. Since NCOA4‐FTH1 has been verified above (Figure 5E; Figure S5A), we conducted co‐IP of BZW1 and NCOA4, and results showed a BZW1‐NCOA4 binding (Figure 6G). Therefore, we conducted co‐IP experiments in CQ‐treated BZW1‐overexpressing cells. Results demonstrated that elevated BZW1 expression could decrease the NCOA4–FTH1 interaction (Figure 6I). PLA assay of anti‐FTH1/NCOA4 showed that NCOA4‐FTH1 interaction could be disrupted by elevated BZW1 expression (Figure 6H). Collectively, these results indicate that BZW1 competitively binds to NCOA4 and subsequently suppresses ferritinophagy.

### 2.7 BZW1 Knockdown Synergizes With Anti‐PD‐1 Therapy Through Ferroptosis‐Mediated Anti‐Tumor Immunity

**FIGURE 7:**
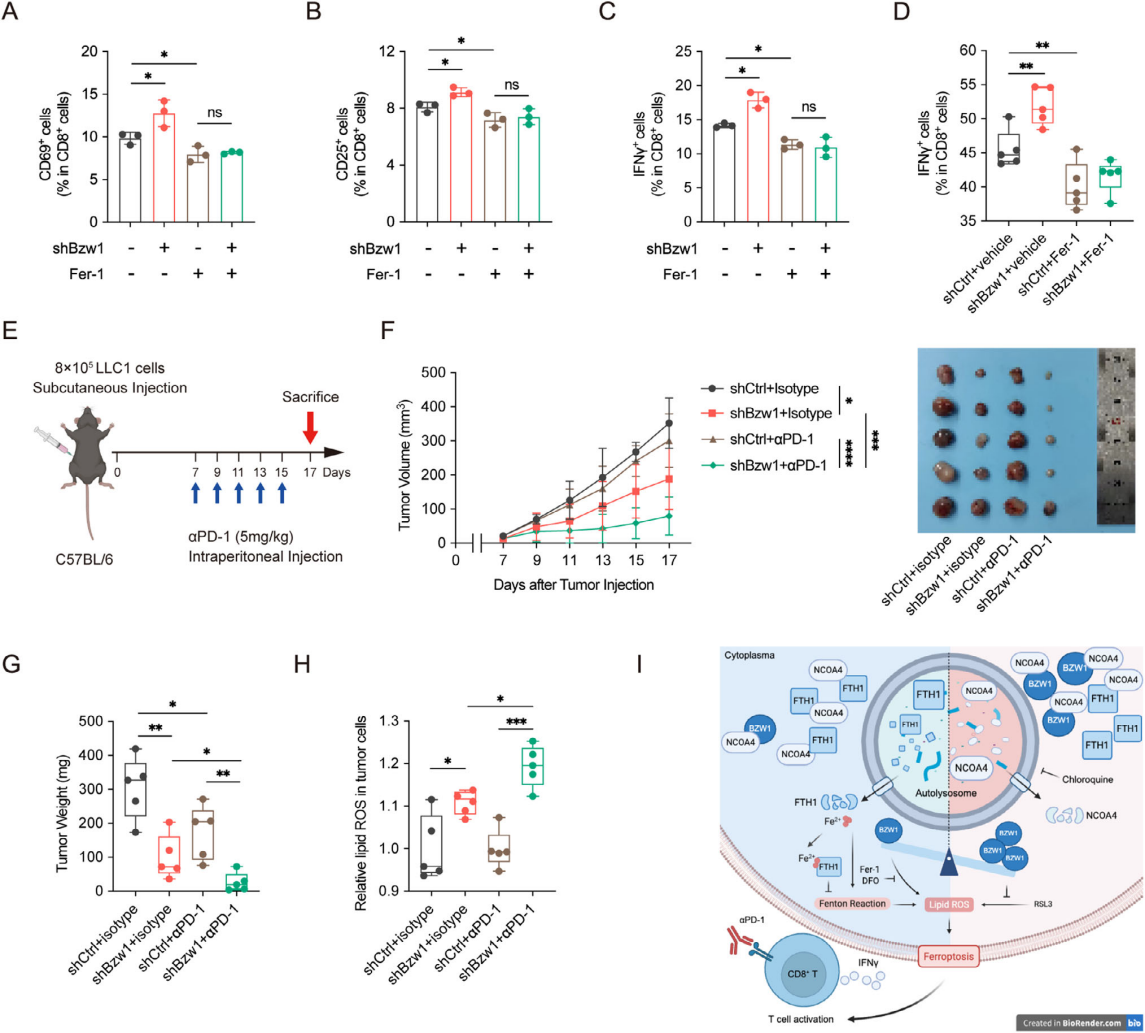
BZW1 targeting synergizes with anti‐PD‐1 therapy through ferroptosis‐mediated immune activation. (A) Proportion of CD69^+^ cells in co‐culture system in indicated treatment groups (n = 3). (B) Proportion of CD25^+^ cells in co‐culture system in indicated treatment groups (n = 3). (C) Proportion of IFN‐γ^+^CD8^+^T cells in co‐culture system in indicated treatment groups (n = 3). (D) Tumor‐infiltrating IFN‐γ^+^CD8^+^T cells in indicated treatment groups (n = 5). (E) Schematic diagram of the therapeutic regimen and experimental timeline. (F) Tumor growth curves and representative tumor images from different treatment groups (n = 5). (G) Tumor weights at experimental endpoint across indicated groups (n = 5). (H) Lipid ROS levels were measured by C11‐BODIPY 581/591 staining in tumors from different treatment conditions (n = 5). (I) Graphic abstract of this study. The scheme was generated in Biorender. Data are presented as mean ± SEM, ^*^ *p* < 0.05, ^**^ *p* < 0.01, ^***^ *p* < 0.001, ^****^ *p* < 0.0001.

To investigate if BZW1 modulates the TIME through ferroptosis, we conducted T cell activation assay in tumor‐splenocytes co‐culture system (Figure 2B), and results showed that Bzw1 knockdown tumor cells could increase the activation of T cells, and this activation effect could be reversed by Fer‐1 administration, indicating that knockdown of Bzw1 promotes T cell activation via ferroptosis (Figure 7A,B). Notably, Fer‐1 administration significantly reduced IFN‐γ^+^ CD8^+^ T cell infiltration in Bzw1‐knockdown tumors in the xenograft, indicating that the enhanced antitumor immunity observed upon BZW1 depletion is ferroptosis‐dependent (Figure 7D). We next established therapeutic models to evaluate the efficacy of combining targeting Bzw1 with immunotherapy (Figure 7E). Results showed that Bzw1 knockdown combined with anti‐PD‐1 treatment markedly inhibited tumor growth compared to either intervention alone (Figure 7F). We further detected the lipid ROS, and results showed an elevated lipid ROS in the Bzw1 knockdown group when combined with anti‐PD‐1 compared to other groups (Figure 7H). All these results demonstrate that BZW1 knockdown could synergize with immunotherapy.

## 3 Discussion and Conclusion

The interplay between immune cells and tumor cells opens up new avenues for understanding cancer biology and cancer immunotherapy [7]. In TIME, cytotoxic CD8+ T cells secrete IFN‐γ and eliminate tumor cells, while tumor cells develop resistance to this cytotoxicity and remodel the TIME. BZW1 has emerged as a significant oncoprotein in multiple malignancies, with its pro‐tumorigenic functions mostly attributed to the promotion of epithelial‐mesenchymal transition and stemness [8, 9, 11]. While bioinformatic studies have suggested a potential link between BZW1 and immune regulation, direct experimental validation has remained absent. Our study now provides multifaceted evidence establishing BZW1 as a clinically relevant oncogene in lung adenocarcinoma, which suppresses ferroptosis and diminishes response to immunotherapy.

Iron metabolism is a core process in ferroptosis, which maintains the intracellular iron homeostasis via the labile iron pool [30]. Ferritin binds with ferrous iron and attenuate Fenton reaction, which induces lipid peroxidases [26]. FTH1 is the major component of ferritin, which contains the conserved domain binding to NCOA4, and this binding is the core process of ferritinophagy [16, 31, 32]. In this study, we found that BZW1 downregulates NCOA4 and attenuates ferritinophagy, thus inhibiting ferroptosis, suggesting BZW1 could be a suppressor of ferroptosis. In the iron homeostasis, NCOA4 expression was governed by the level of intracellular Fe^2+^. In this process, HERC2 serves as an E3 ubiquitin ligase for NCOA4, and under excess iron conditions, the binding of HERC2 and NCOA4 could be enhanced, thus leading to NCOA4 degradation [33]. In addition, HERC2‐mediated NCOA4 ubiquitin‐mediated degradation was discovered not to be the sole degradation of NCOA4, and NCOA4 ungergoes other degradation [34]. Previous studies have also revealed that the NCOA4‐FTH1 binding could be disrupted through other bonds, and results have shown that both NCOA4 and FTH1 could bind with other compounds or molecules [19, 20]. In our study, we observed a direct binding of BZW1 and NCOA4, and the binding of NCOA4 and FTH1 is decreased when BZW1 is overexpressed. Since no binding of BZW1 and FTH1 was detected, our results demonstrate that BZW1 could disrupt the NCOA4‐FTH1 complex via a competitive binding with NCOA4; however, the mechanism of this competitive binding needs to be further explored. Our study found that BZW1 could promote the autophagic degradation of NCOA4, but whether this binding results in autophagy still needs to be explored.

Emerging studies have shown that targeting tumor ferroptosis is a promising anti‐cancer strategy [24, 35, 36]. Several ferroptosis inducers, such as erastin and RSL3, have shown suppressive effects on tumors in murine models [21, 22]. Moreover, accumulating evidence suggests a strong link between ferroptosis and tumor immune microenvironment, which shed lights on the combination of ferroptosis inducer and ICBs [23, 37, 38, 39]. Previous studies have shown that ferroptotic cancer cells could modulate immune responses in the TIME [21, 22, 39]. In TIME, although cytotoxic CD8^+^T cells are the primary effector in anti‐tumor immunity, the interaction between ferroptotic tumor cells and TIME affects other immune cells as well, such as Th1 cells, macrophages, DC cells, and MDSCs [40, 41, 42]. Preclinical studies have shown that targeting ferroptosis can sensitize tumors to immunotherapy, which provides novel aspects in combined therapy.

Our findings demonstrate that BZW1 fosters an immunosuppressive tumor microenvironment via ferroptosis suppression. Specifically, BZW1‐knockdown tumors exhibited enhanced lipid peroxidation and ferroptosis, promoted increased CD8^+^T cell infiltration while reducing immunosuppressive populations. Mechanistically, BZW1 promotes autophagic degradation of the ferritinophagy receptor NCOA4 and competitively binds to NCOA4 to disrupt the NCOA4–FTH1 interaction. This dual action stabilizes ferritin, restricts intracellular Fe^2+^ release, and ultimately inhibits ferroptotic cell death. In the tumor microenvironment, BZW1 ablation promotes ferroptosis‐driven immunogenic cell death, leading to enhanced activation and infiltration of cytotoxic T cells. The consequent transformation from an immunologically “cold” to “hot” tumor microenvironment significantly enhanced sensitivity to immunotherapy. Our findings nominate BZW1 as a promising prognostic biomarker and a therapeutic target whose inhibition may sensitize NSCLC to immune checkpoint blockade.

## 4 Experimental Section

### 4.1 Cell Culture

The human lung cancer cell lines A549, NCI‐H1299, NCI‐H1975, NCI‐H1650, NCI‐H1703, NCI‐H2170, and SK‐MES‐1, the mouse Lewis lung carcinoma (LLC) cell line, and the human embryonic kidney cell line HEK293T were acquired from the Cell Bank of the Chinese Academy of Sciences (Shanghai, China). All cells were maintained at 37 °C in a humidified 5% CO_2_ atmosphere and passaged using 0.25% trypsin. The culture media were supplemented with 10% fetal bovine serum (FBS), 100 U/mL penicillin, and 0.1 mg/mL streptomycin. Specifically, LLC and HEK293T cells were cultured in Dulbecco's modified Eagle's medium (DMEM). A549, NCI‐H1299, NCI‐H1975, NCI‐NCI‐H1650, NCI‐H1703, and NCI‐H2170 cells were grown in RPMI‐1640 medium. SK‐MES‐1 cells were maintained in MEM medium.

### 4.2 Lentivirus Production and Infection

Stable knockdown of human BZW1 was achieved using the pLKO.1‐U6‐Puro vector (Mailgene, Beijing) carrying either control (shCtrl) or BZW1‐targeting (shBZW1) shRNA sequences. Stable knockdown of mouse Bzw1 was performed using pLKO.1‐U6‐Puro backbone with corresponding shCtrl or shBzw1 constructs, and stable murine Ncoa4 knockdown was achieved using the pLVX‐IRES‐NEO vector (Tsingke Biotech, China) carrying control (shNC) or Ncoa4‐targeting (shNcoa4) shRNAs. The overexpression plasmid for human BZW1 was synthesized by Mailgene using the pCDH‐U6‐Puro vector, and the overexpression plasmids for human FTH1 and mouse Fth1 were synthesized by Tsingke Biotech using the pLVX‐IRES‐NEO vector. Lentiviruses were packaged in HEK293T cells using the second‐generation system comprising psPAX2 (#12260, Addgene) and pMD2.G (#12259, Addgene). Transfection was performed with Hieff Trans Liposomal Transfection Reagent (#40802, Yeasen, Shanghai) following the manufacturer's instructions. Target cells underwent two rounds of lentiviral transduction in the presence of 8 µg/mL polybrene (H9268, Sigma‐Aldrich) over 48 h. Stable transductants were selected with 2 µg/mL puromycin (A610593, Sangon Biotech) or 0.5 mg/mL G418 (A600958, Sangon Biotech) for 7 days. All shRNA sequences used in this study are provided in Table S1.

### 4.3 Reagents

All chemical compounds were obtained from MedChemExpress, including Z‐VAD (HY‐164388), Necrostatin‐1 (HY‐15760), Ferrostatin‐1 (HY‐100579), RSL3 (HY‐100218A), Deferoxamine (HY‐B1625), Cycloheximide (HY‐12320), MG‐132 (HY‐1325), and Chloroquine (HY‐17589A).

### 4.4 Cell Viability Assay

For the CCK‐8 assay, approximately 5 × 10^3^ cells transfected with the indicated plasmids or shRNAs were seeded into 96‐well plates and cultured for 24 h. Following incubation, the medium was replaced with 100 µL of serum‐free medium containing 10% CCK‐8 reagent (TargetMol, Shanghai, China). After an additional 1 h incubation at 37°C, the optical density (OD) at 450 nm was measured using a microplate reader.

### 4.5 Western Blot

Whole‐cell lysates were prepared using RIPA lysis buffer (New Cell & Molecular Biotech Co., Ltd., Suzhou, China) supplemented with a protease inhibitor cocktail (Thermo Scientific Technologies, USA). Protein concentrations were determined with a Bradford assay kit (Thermo Scientific Technologies, USA). For Western blot analysis, the following primary antibodies were used: anti‐BZW1 (19917‐1‐AP, Proteintech, 1:5000), anti‐NCOA4 (83394‐4‐RR, Proteintech, 1:8000), anti‐FTH1 (A19544, Abclonal, 1:10000), and anti‐GAPDH (60004‐1‐Ig, Proteintech, 1:5000). GAPDH served as the loading control. For co‐immunoprecipitation (co‐IP) assays, 2 µg of anti‐BZW1 (85655‐1‐RR, Proteintech), anti‐NCOA4 (A25307, Abclonal), or normal rabbit IgG (2729S, Cell Signaling Technology) were conjugated to protein A/G magnetic beads (HY‐K0202, MedChemExpress) by incubation at 4°C for 6–8 h. Cells were lysed in IP lysis buffer (P0013, Beyotime Biotechnology) containing protease inhibitors (Thermo Scientific Technologies, USA). Equal amounts of protein lysates were incubated with the antibody‐conjugated beads overnight at 4°C. Beads were subsequently washed four times with PBS containing 0.5% Triton X‐100, and immunoprecipitated proteins were analyzed by western blotting.

### 4.6 RNA Isolation and Quantitative Real‐Time PCR (qRT‐PCR)

Total RNA was extracted using the SteadyPure Quick RNA Extraction Kit (AG21023, Accurate Biotechnology), and cDNA synthesis was performed with the FastKing RT Kit (TIANGEN BIOTECH, Beijing) following the manufacturer's protocols. Quantitative real‐time PCR (qRT‐PCR) was conducted using PowerUpTM SYBR Green Master Mix (A25742, Applied Biosystems, CA, USA) on a StepOnePlus Real‐Time PCR System (Applied Biosystems, CA, USA). Gene expression levels were normalized to GAPDH as an internal control, and the primer sequences used are provided in Table S2.

### 4.7 Histology and Immunohistochemistry

Human lung cancer tissue microarrays (TMAs) containing 180 cases were obtained from the Cancer Hospital of the Chinese Academy of Medical Sciences between March and May 2024. Human and mouse tumor tissues were fixed in 4% paraformaldehyde, embedded in paraffin, and sectioned using a microtome. For immunohistochemistry, tissue sections were blocked with goat serum for 1 h and incubated overnight at 4°C with the following primary antibodies: anti‐BZW1 (19917‐1‐AP, Proteintech; 1:200), anti‐NCOA4 (10968‐1‐AP, Proteintech; 1:300), anti‐FTH1 (A19544, Abclonal; 1:300) and anti‐Ki‐67 (GB121141, Servicebio; 1:300). Signal detection was performed using appropriate secondary antibodies, and nuclei were counterstained with hematoxylin (ZSGB‐BIO).

### 4.8 Immunofluorescence (IF)

Approximately 10^4^ cells transfected with the indicated plasmids or shRNAs were seeded into 24‐well plates and allowed to adhere. Following fixation with 4% paraformaldehyde and permeabilization with 0.5% Triton X‐100, cells were blocked with 5% bovine serum albumin. Primary antibodies against BZW1, NCOA4, and FTH1 were conjugated using labeling kits (KFA009, KFA001, KFA003; Proteintech) according to the manufacturer's instructions. Immunostaining was performed by incubating cells with the conjugated antibodies overnight at 4 °C. Nuclei were counterstained with DAPI, and images were acquired using a confocal fluorescence microscope (Nikon, Japan).

### 4.9 Transmission Electron Microscopy (TEM)

For ultrastructural analysis, tissue specimens were sequentially processed as follows: initial embedding in 10% gelatin was followed by fixation with 2.5% glutaraldehyde at 4°C for 24 h. Fixed samples were then trimmed into <1 mm^3^ blocks and dehydrated through a graded ethanol series (30%, 50%, 70%, 90%, 95%, and 100%), with 10 min incubations at each concentration. Samples were subsequently infiltrated with Quetol‐812 epoxy resin progressively diluted in propylene oxide (25%, 50%, 75%, and 100%), with 3 h intervals at each concentration. Following infiltration, specimens were embedded in pure Quetol‐812 resin and polymerized under sequential temperature conditions: 35°C (12 h), 45°C (12 h), and 60°C (24 h). Ultrathin sections (100 nm) were prepared using a Leica UC6 ultramicrotome, double‐stained with uranyl acetate (10 min) and lead citrate (5 min) at room temperature, and examined using an FEI Tecnai T20 transmission electron microscope operated at 120 kV.

### 4.10 Assessment of Lipid Peroxidation by BODIPY‐C11 Staining

Cells were incubated with 1 µm BODIPY 581/591 C11 (Beyotime Biotechnology, Shanghai, China) at 37°C for 30 min under light‐protected conditions. After incubation, cells were washed twice with cold PBS and resuspended in 200 µL PBS for immediate analysis. Flow cytometry was performed using an ID7000 Spectral Cell Analyzer (Sony Biotechnology, Japan), with the non‐oxidized form detected in the PE channel (excitation/emission: 581/591 nm) and the oxidized form detected in the FITC channel (excitation/emission: 488/510 nm). The degree of lipid peroxidation was quantified as the ratio of FITC mean fluorescence intensity (MFI) to PE MFI, with all results normalized to control samples to determine relative lipid ROS levels.

### 4.11 Evaluation of Intracellular Fe^2+^ Levels

FerroOrange probe (Dojindo Laboratories, Japan) was used for intracellular Fe^2+^ levels evaluation. Indicated cells were incubated with 1 µM FerroOrange at 37°C for 20 min and subsequently analyzed by flow cytometry. The mean fluorescence intensity (MFI) in the PE channel was quantified for each sample. To ensure comparability across experiments, Fe^2+^ levels were normalized to control samples and expressed as relative fold changes.

### 4.12 Proximity Ligation Assay (PLA)

PLA was conducted using Duolink In Situ PLA kit (DUO92001, Sigma–Aldrich) according to the manufacturer's protocol, and images were acquired using a Super‐Resolution Microscopy (GE, USA).

### 4.13 Animals

All animal experiments were conducted in accordance with protocols approved by the Animal Care and Use Committee of the Chinese Academy of Medical Sciences Cancer Hospital (NCC2024A309). Subcutaneous xenograft models were established using five‐week‐old male C57BL/6J and Balb/c nude mice (Charles River Laboratories, Beijing, China). Mice were randomly assigned to experimental groups with a minimum sample size of five animals per group. For the H1299 xenograft model, Balb/c nude mice were subcutaneously injected with 2 × 10^6^ H1299 cells stably transfected with either an empty vector or a BZW1‐overexpressing plasmid. In the LLC syngeneic model, C57BL/6J mice received subcutaneous injections of 8 × 10^5^ LLC cells transfected with corresponding constructs. For ferroptosis inhibition studies, Ferrostatin‐1 (Fer‐1) was reconstituted according to manufacturer specifications and administered intraperitoneally every two days, beginning on day 6 post‐inoculation; control animals received vehicle only. For Ncoa4 knockdown groups, LLC cells were stably transfected with corresponding shRNAs before the implantation. For immunotherapy assessment, an anti‐PD‐1 antibody or isotype control was administered intraperitoneally every two days starting from day 7 post‐inoculation. Tumor volumes were measured periodically and calculated using the formula: (width [2] × length)/2. At experimental endpoints, tumors were harvested for subsequent immunohistochemical analysis or flow cytometry.

### 4.14 siRNA Transfection

Cells were transfected with siRNA using Lipofectamine 3000 (Invitrogen) according to the manufacturer's instructions. All siRNA sequences used in this study are provided in Table S3.

### 4.15 Flow Cytometry

For TILs analysis, the freshly harvested tumor was minced and digested with Collagenase Type IV solution (1 mg/mL; #C9263‐1G, Sigma–Aldrich) and DNase I (20 µg/mL; #10104159001, Roche, Switzerland) in a constant temperature shaker at 37°C for 30 min. Digestion was terminated by adding FBS‐supplemented medium, and the suspension was filtered through a 100 µm strainer. After red blood cell lysis, cells were washed and resuspended in PBS adjusted to an appropriate concentration. For surface and intracellular marker staining, cells were first blocked with purified mouse anti‐CD16/32 (#70‐0161‐M001, Tonbo Biosciences, USA), followed by Zombie Aqua Fixable viability kit (#423101, BioLegend, USA) according to the manufacturer's instructions. Surface staining was performed in PBS containing 1% BSA and incubated with surface antibodies for 30 min. For cytokine staining, cells were treated with Cell Activation Cocktail (with Brefeldin A)(#42330, BioLegend). After activation, cells were fixed and permeabilized using the Intracellular Fixation & Permeabilization Buffer Kit (88‐8824‐00, eBioscience). Antibodies utilized for this section are detailed in the Supporting Information.

### 4.16 RNA Sequencing and Bioinformatics Analysis

RNA sequencing was conducted by Sequanta Technologies Co., Ltd (Shanghai, China). Three biologically independent replicates were prepared per group. Paired‐end libraries were constructed using the NEBNext UltraTM RNA Library Prep Kit (New England Biolabs, USA) and sequenced on the Illumina NovaSeq 6000 platform. Differentially expressed genes were identified using thresholds of |log_2_(fold change)| > 1 and adjusted p‐value < 0.05.

### 4.17 Proteomic Sequencing and Analysis

Proteomic Sequencing was conducted by Personal Biotechnology Co., Ltd (Shanghai, China). Proteins with fold change >1.5 and FDR < 0.05 were considered differentially expressed. Functional enrichment analysis was conducted using clusterProfiler (v4.6.2).

### 4.18 Statistical Analysis

The data were analyzed using GraphPad Prism 10 (GraphPad Software, CA, USA). Unpaired or paired Student's t test, two‐way ANOVA, and the Kaplan‐Meier method were used for comparisons between two groups, comparisons among multiple groups, and survival analysis, respectively. Statistical significance was assumed for p‐values ≤ 0.05, and results from at least three biologically independent experiments with similar results are reported. The data are presented as the mean ± s.e.m. or mean ± s.d. values.

## Author Contributions

S.G. and Z.L. assisted in concept and design; Y. Li and L. Ma assisted in data collection, analysis, and interpretation; M. Hu and S. Zheng assisted in writing, reviewing, and/or revising manuscripts; S. Liu assisted in administrative, technical, or material support.

## Funding

This work was supported by the National High‐Level Hospital Clinical Research Funding (2025‐LYZX‐C‐A02), the National Key R&D Program of China (2021YFC2500900), the National Natural Science Foundation of China (82273129), the Central Health Research Key Projects (2022ZD17), and the CAMS Innovation Fund for Medical Sciences (CIFMS)(2021‐I2M‐1‐015, 2024‐I2M‐C&T‐C‐008, and 2024‐I2M‐ZH‐005).

## Conflicts of Interest

The authors declare no conflict of interest.

## Supporting information




**Supporting File**: advs74801‐sup‐0001‐SuppMat.docx.

## Data Availability

The data that support the findings of this study are available from the corresponding author upon reasonable request.
