# Supplementary material for: BZW1 Drives Immune Evasion in Lung Adenocarcinoma via Ferroptosis Suppression
Source: Adv Sci (Weinh). 2026 Mar 15;13(29):e21885. doi: 10.1002/advs.202521885 (PMC13205648; doi:10.1002/advs.202521885)
Supplement: Supplementary file 1 — Supporting File: advs74801‐sup‐0001‐SuppMat.docx. [file ADVS-13-e21885-s001.docx]

Supporting Information

**BZW1 Drives Immune Evasion in NSCLC by Competitively Binding NCOA4 to Suppress Ferritinophagy-Dependent Ferroptosis**

Zhao Linyao, Peng Yue, Liang Qing, Liu Shi, Li Yang, Ma Lei, Hu Menghan, Zheng Sujuan, Liu Zhihua*, Gao Shugeng*

**Supporting information experiment section**

*Colon Formation Assay:* Approximately 1×10^3^ cells transfected with corresponding plasmids or shRNAs were seeded into a 6-well plate and the culture medium was replenished every two days. Cells were cultured for 10-14 days until colonies became visible to naked eyes. Medium was removed and cells were washed twice with PBS. Colonies were fixed with 4% paraformaldehyde for 15 min at room temperature (RT), rinsed with PBS, and stained with 0.5% crystal violet (Sigma-Aldrich) in methanol for 30 min at RT. Plates were washed gently under running tap water and air-dried overnight.

*Cell viability assay*: For cell viability assay, 8×10^3^ H1299 and A549 cells were seeded into 96-well plates. After RSL3 treatment for 24 h and 48 h, CCK-8 reagent (TargetMol, Shanghai, China) was added to the serum-free medium at a ratio of 1:10, and the absorbance was measured using a microplate reader (BioTek, VT, USA) at 450 nm after incubation for 1 h at 37°C.

**Supporting information figures**

**
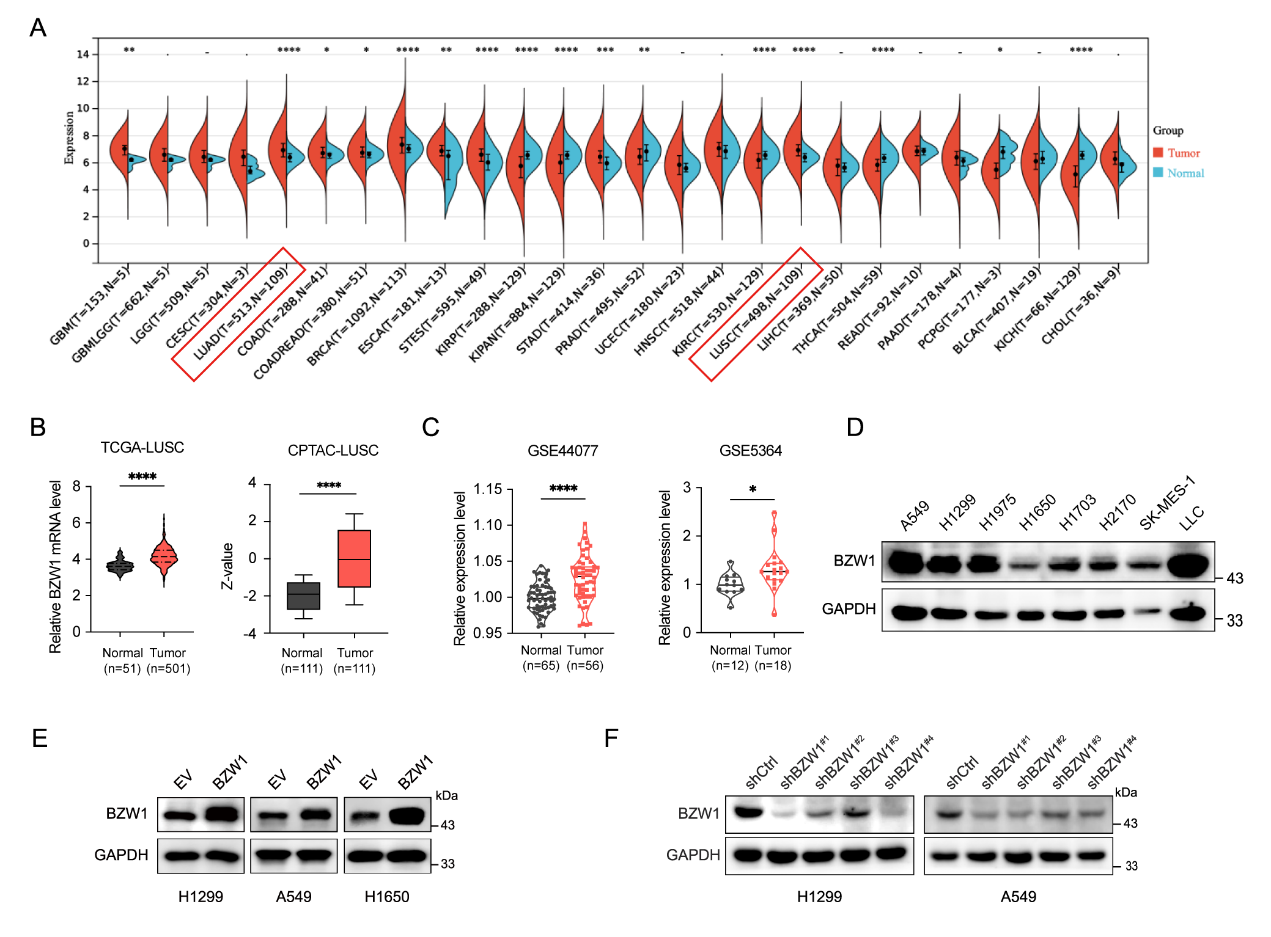
**

**Figure S1. Pan-cancer and large-scale cohort analysis of BZW1 expression patterns.** A) BZW1 expression levels in TCGA pan-cancer atlas. B) BZW1 expression in LUSC in TCGA and CPTAC. C) BZW1 expression in large-scale lung cancer cohorts. D) Western blot analysis of BZW1 expression levels in different lung cancer cell lines. E) Western blot validation of BZW1 overexpression in stable transfected cell lines. F) BZW1 knockdown efficiency in lung cancer cells. The data are presented as the mean ± s.e.m. *p < 0.05, **p < 0.01, ***p < 0.001, ****p < 0.0001.

**
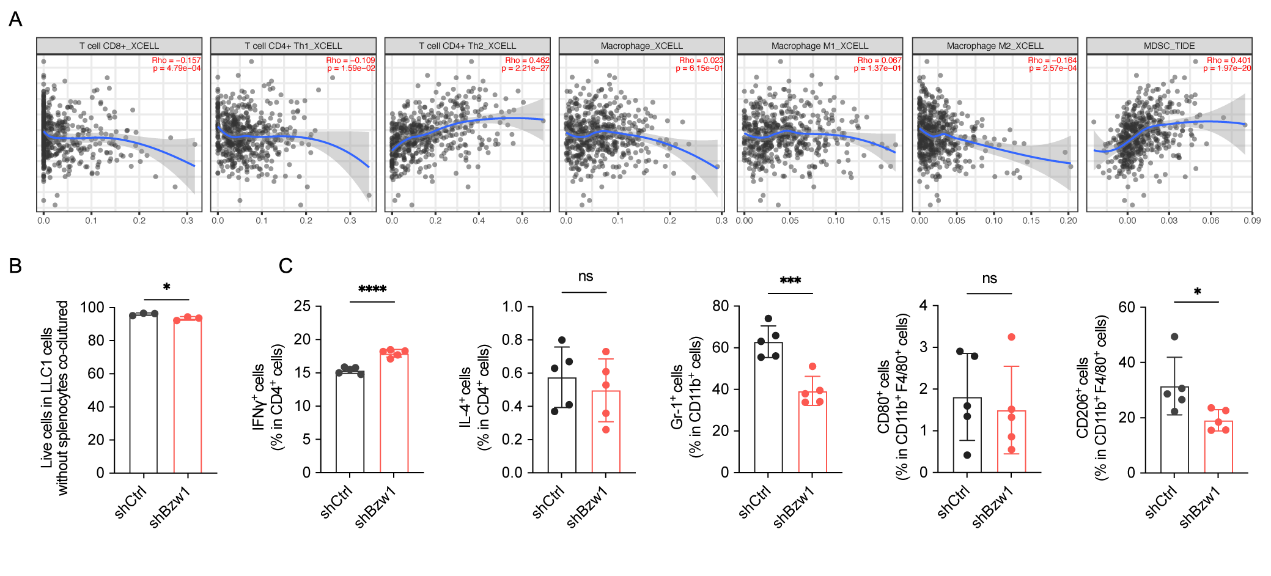
**

**Figure S2. Bioinformatic analysis and immune infiltration analysis of BZW1 expression and tumor microenvironment.** A) Bioinformatic analysis of BZW1 expression and immune infiltration in TCGA-LUAD. B) Live tumor cells after BZW1 knockdown (n=3). C) Intratumor immune infiltration in Bzw1 knockdown group (n=5). The data are presented as the mean ± s.e.m. *p < 0.05, ***p < 0.001, ****p < 0.0001.

**
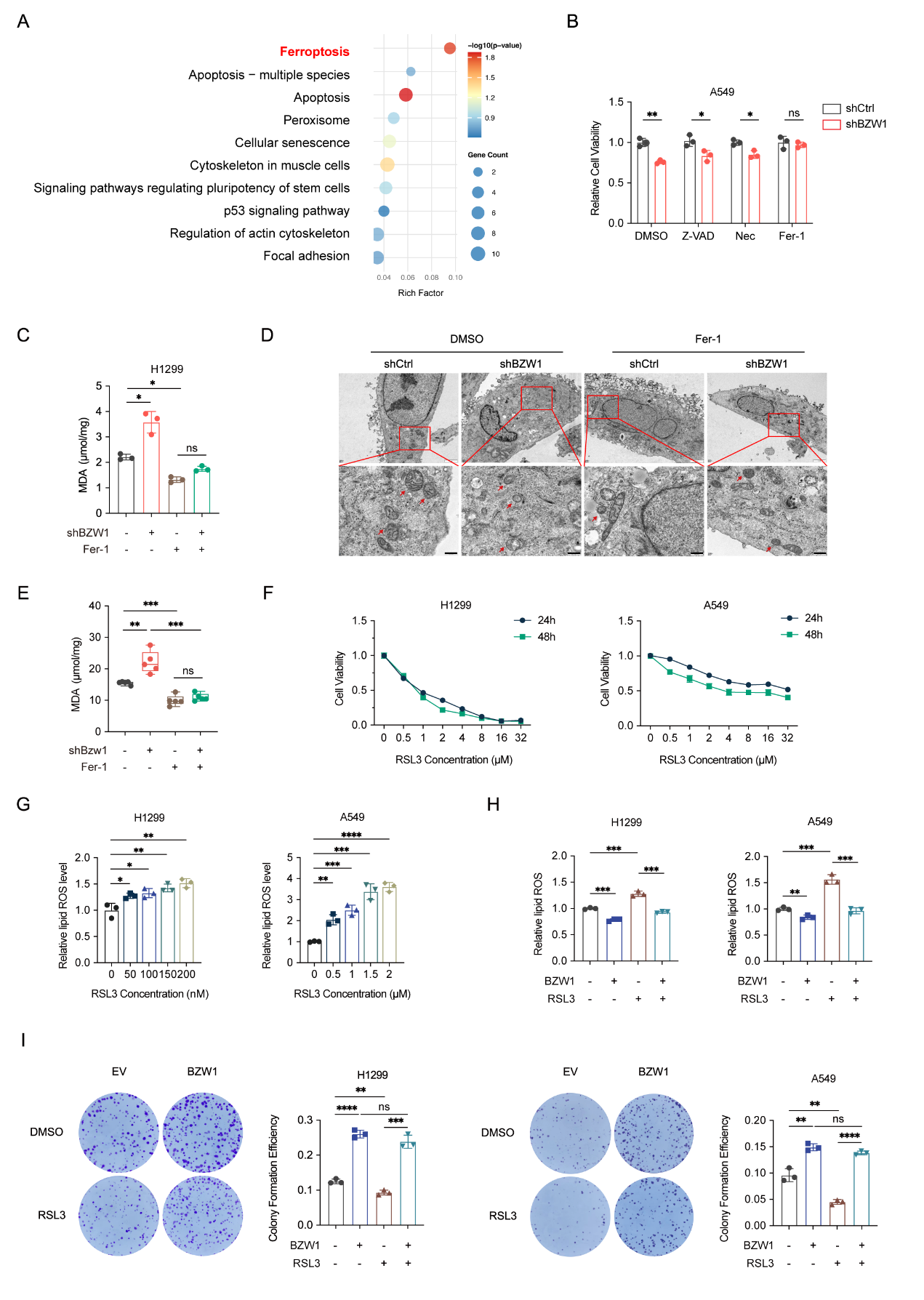
**

**Figure S3. BZW1 suppresses ferroptosis in lung cancer cells.** A) KEGG pathway enrichment of RNA-seq derived differentially expressed genes (DEGs) in BZW1-knockdown H1299 cells. B) Cell viability of BZW1-knockdown cells treated with Z-VAD (20 µM), necrostatin-1 (Nec-1, 20 µM), or ferrostatin-1 (Fer-1, 20 µM), assessed by CCK-8 assay (n=3). C) MDA level in BZW1 knockdown cells with Fer-1 (n=3). D) Representative images of TEM in A549 cells. Scale bar: 100nm. E) MDA level in Bzw1 knockdown tumor administrated with Fer-1 (n=5). F) Cell viability was measured in different concentration of RSL3 in lung cancer cells (n=3). G) Lipid peroxidation levels was assessed via C11 BOPIDY 581/591 staining (n=3). H) Lipid peroxidation levels was assessed via C11 BOPIDY 581/591 staining in indicated groups (n=3). I) Representative images of colony formation assay and quantitative analysis of BZW1-overexpressing cells treated with RSL3 (n=3). The data are presented as the mean ± s.e.m. *p < 0.05, **p < 0.01, ***p < 0.001, ****p < 0.0001.

**
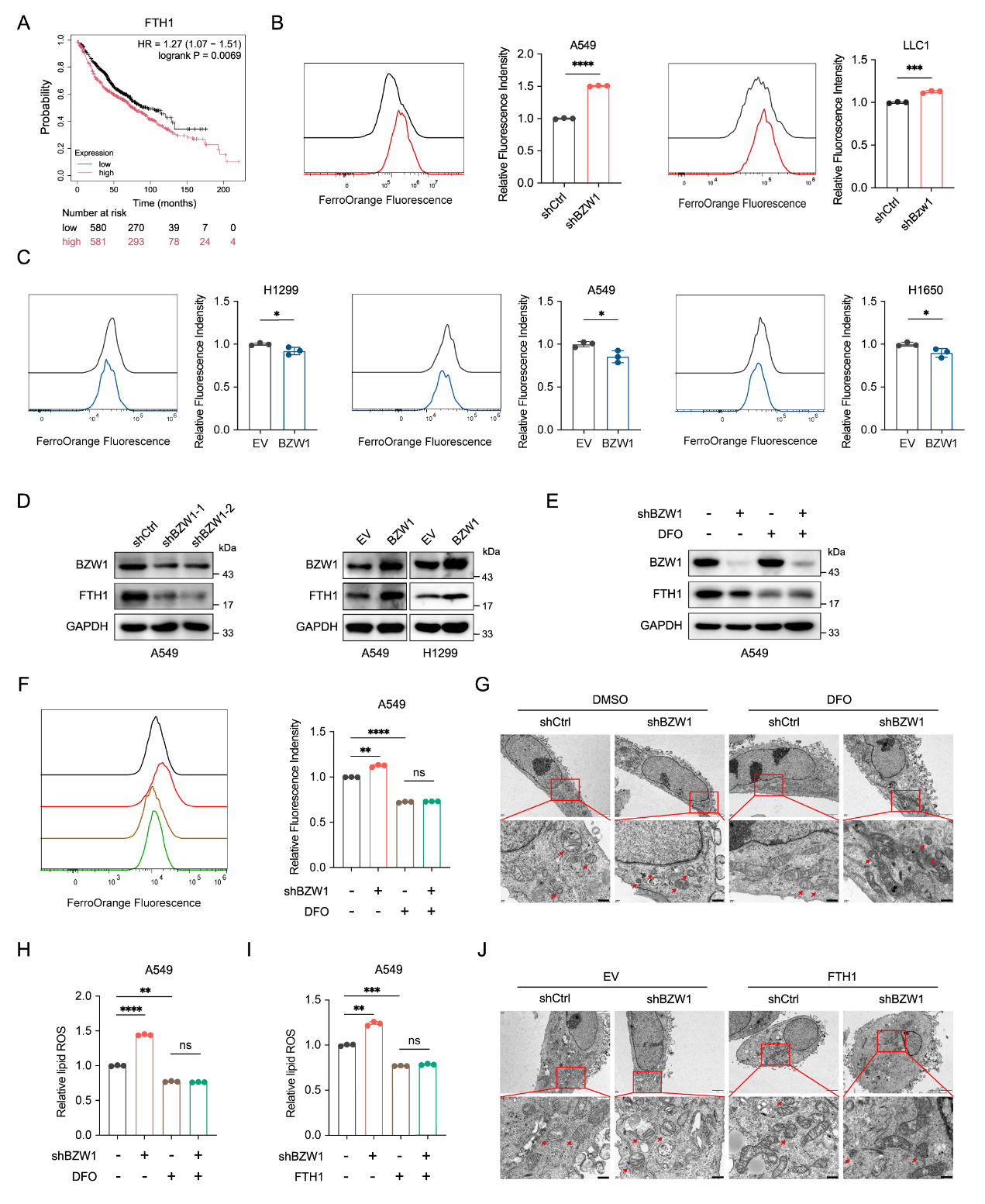
**

**Figure S4. BZW1 regulates ferroptosis through modulation of the labile iron pool.** A) Kaplan-Meier survival analysis of lung cancer patients stratified by FTH1 expression levels. B,C) Intracellular Fe²^+^ levels measured by FerroOrange staining in indicated experimental groups (n=3). D,E) Western blot analysis of FTH1 protein expression in the indicated groups. F) Lipid peroxidation levels was assessed via C11 BOPIDY 581/591 staining in indicated groups (n=3). G) Representative images of TEM in A549 cells. Scale bar: 100nm. H,I) Lipid peroxidation levels was assessed via C11 BOPIDY 581/591 staining in indicated groups (n=3). J) Representative images of TEM in A549 cells. Scale bar: 100nm. The data are presented as the mean ± s.e.m. **p < 0.01, ***p < 0.001, ****p < 0.0001.

**
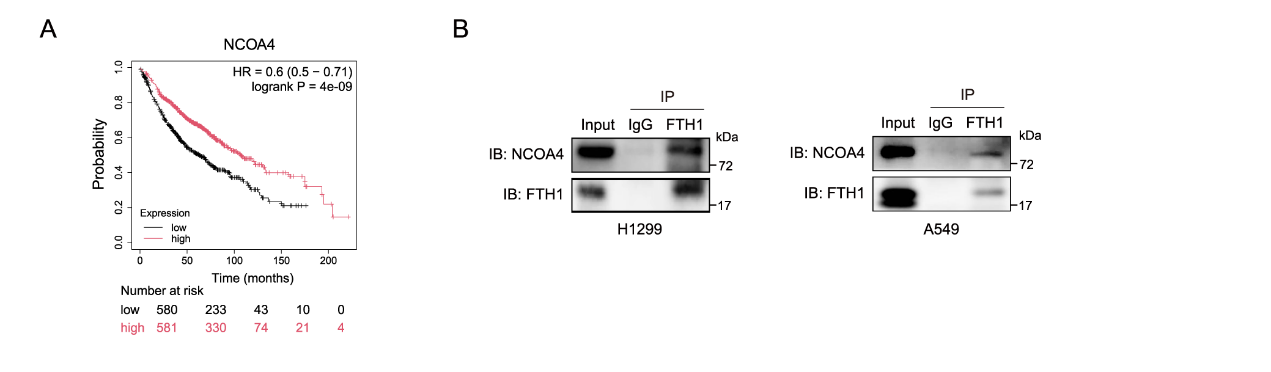
**

**Figure S5. BZW1 modulates the labile iron pool through ferritinophagy regulation.** A) Kaplan-Meier survival analysis of lung cancer patients stratified by NCOA4 expression levels. B) Co-IP assays analyzing FTH1-NCOA4 protein interactions.


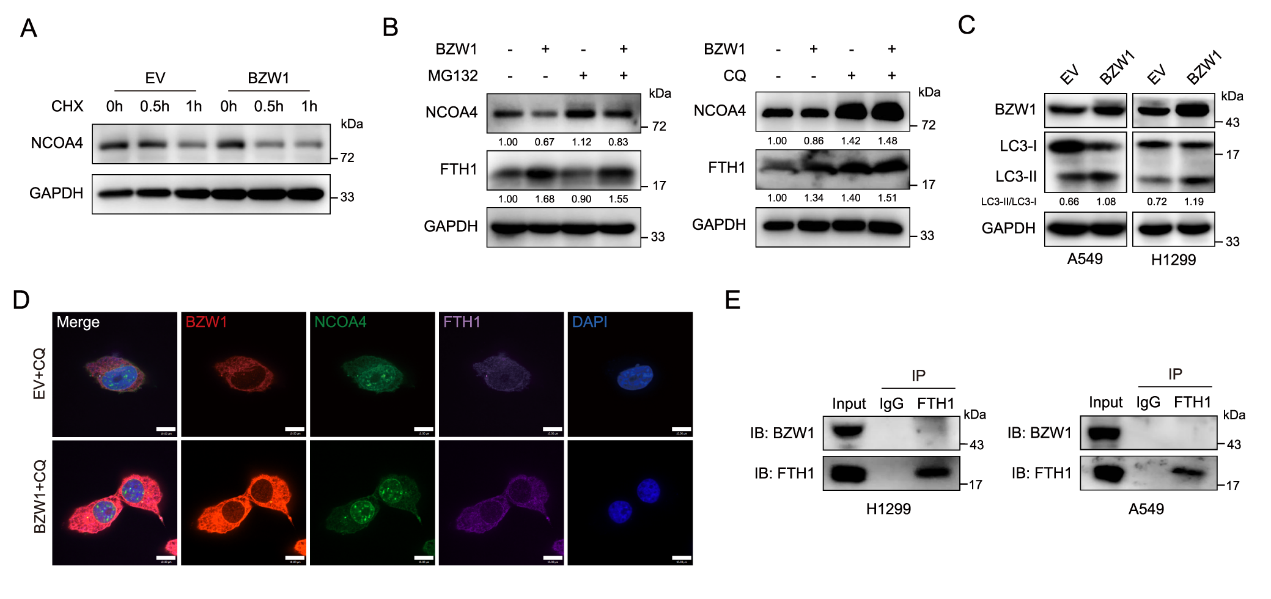


**Figure S6. BZW1 disrupts the NCOA4-FTH1 complex through direct binding to NCOA4. A)** Western blot analysis of NCOA4 protein degradation kinetics in A549 cells treated with cycloheximide (100 μg/mL). B) NCOA4 protein stability assessment in cells treated with MG132 (10 μM) and chloroquine (CQ, 50 μM). C) Western blot analysis of LC3B in indicating groups. D) Immunofluorescence imaging of BZW1 (red), NCOA4 (green), FTH1 (purple), and DAPI-stained nuclei (blue) in CQ-treated cells (50 μM). Scale bar: 10 μm. E) Co-IP analysis of interaction between BZW1 and FTH1.


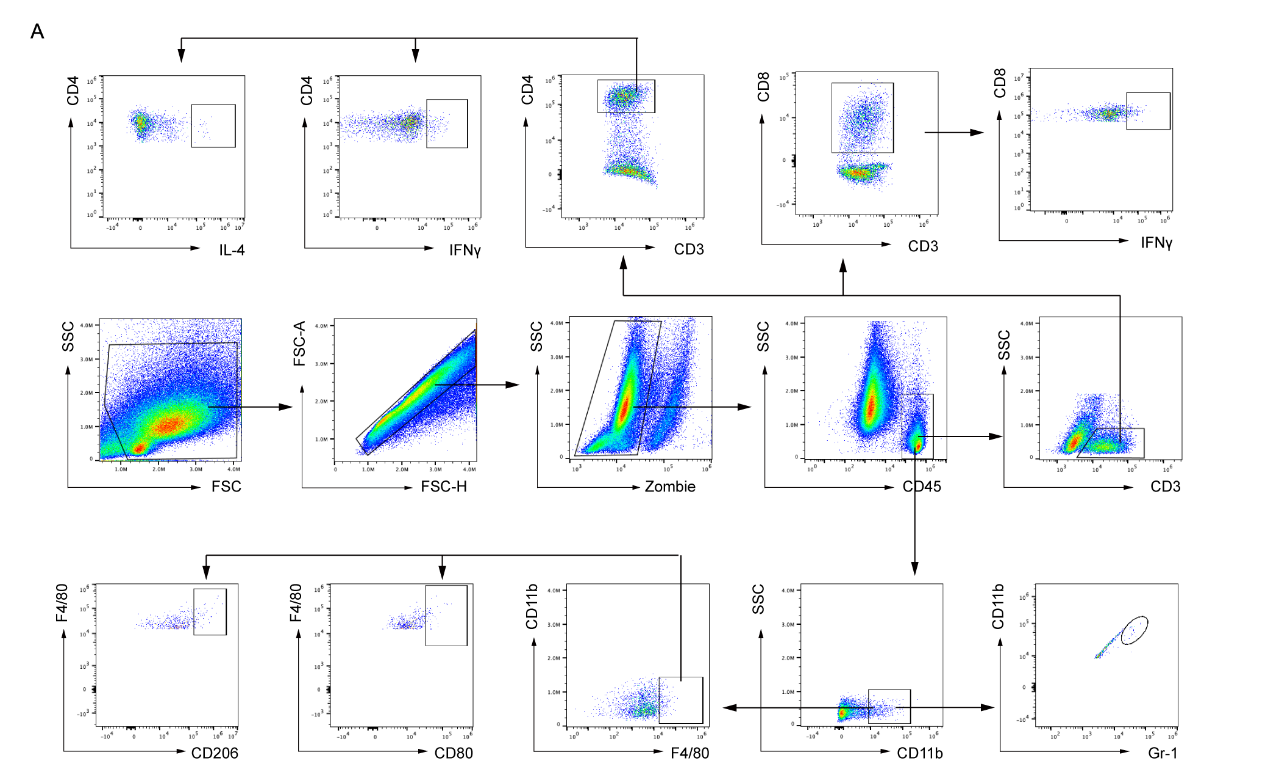


**Figure S7. Gating strategy for flow cytometry.** Gating strategy for flow cytometry to identify distinct cell populations in mouse tumor or spleen. CD4^+^ T cells (CD3^+^, CD4^+^), CD8^+^ T cells (CD3^+^, CD8^+^), Macrophages (CD11b^+^, F4/80^+^), M1-like macrophages (CD11b^+^, F4/80^+^, CD80^+^), M2-like macrophages (CD11b^+^, F4/80^+^, CD206^+^), IFN-γ^+^ CD8^+^ T cells (CD3^+^, CD8^+^, IFN-γ+) gated on CD45+ cells.

**Table S1.** Sequences of shRNAs used in this study.

| Target Gene | Vector | shRNA Name | Sequence (5′→3′) |
| --- | --- | --- | --- |
| Human BZW1 | pLKO.1-U6-puro | shBZW1-1 | ACTGAAACCGGTACTGATTTG |
|  | pLKO.1-U6-puro | shBZW1-2 | GTACAGATGTCTGCGTGTTTG |
| Mouse Bzw1 | pLKO.1-U6-puro | shBzw1-1 | CCTTAATGCATCCATTCTTAA |
|  | pLKO.1-U6-puro | shBzw1-2 | ACTGTATTATTCAAGGCTTAA |
| Mouse Ncoa4 | pLVX-U6-Neo | shNcoa4 | GCAGCTTAAGGTTGATAAA |

**Table S2.** Primer Sequences for qRT-PCR Analysis.

| Gene Name | Species | Sequence (5′→3′) |
| --- | --- | --- |
| BZW1 | Human | F:TGCAGTAGCTGCAAGTCTTCGG  R:CTCCGATGGTTTGCTGATTCCG |
| NCOA4 | Human | F:GAGGTGTAGTGATGCACGGAG  R:GACGGCTTATGCAACTGTGAA |
| FTH1 | Human | F:TGAAGCTGCAGAACCAACGAGG  R:GCACACTCCATTGCATTCAGCC |
| GAPDH | Human | F:GTCTCCTCTGACTTCAACAGCG  R:ACCACCCTGTTGCTGTAGCCAA |

**Table S3.** Sequences of siRNAs used in this study.

| Gene Name | Species | siRNA Name | Sequence (5′→3′) |
| --- | --- | --- | --- |
| NCOA4 | Human | si-NCOA4 | Sense:CCAGGAAGUAUUACUUAAU  Antisense:AUUAAGUAAUACUUCCUGG |
